# Species Composition, Distribution and Habitat Types of Odonata in the iSimangaliso Wetland Park, KwaZulu-Natal, South Africa and the Associated Conservation Implications

**DOI:** 10.1371/journal.pone.0092588

**Published:** 2014-03-24

**Authors:** Lorinda A. Hart, Meyrick B. Bowker, Warwick Tarboton, Colleen T. Downs

**Affiliations:** School of Life Sciences, University of KwaZulu-Natal, Pietermaritzburg, South Africa; Onderstepoort Veterinary Institute, South Africa

## Abstract

Maputaland–Pondoland–Albany, South Africa has been identified as a biodiversity hotspot and centre for endemism. Odonata make good indicators of freshwater ecosystem health. Consequently we compiled a list of Odonata species recorded to date in the iSimangaliso Wetland Park. We then detailed important species in terms of endemism, conservation status, and potential as indicator species. Finally, we compared Odonata assemblages of different sites sampled within the park to illustrate habitat importance. Species identified during two formal surveys and incidental observations made during the study period were combined with an existing database to compile an accurate and up to date species list for the iSimangaliso Wetland Park. Data from this study were then analyzed to determine which water bodies had the most similar species composition. The Dragonfly Biotic Index (DBI) value of each study area was also determined. We recorded 68 odonate species in the iSimangaliso Wetland Park, adding 13 species to the Ezemvelo KwaZulu-Natal Wildlife database for the area. This brings the total number of Odonata species for the iSimangaliso Wetland Park to 86. Eight species are red-listed, 12 are restricted in South Africa to the coastal plains of northern KwaZulu-Natal, and the remainder occurs widely across the southern African savanna. Analyses indicate that species odonate assemblages were most similar in water bodies with comparable habitats. iSimangaliso Wetland Park is identified as an important area for Odonata diversity and endemism, a trend also reflected by the DBI values. Shifts in the existing species assemblages would indicate changes within the ecosystem and thus this species account provides necessary baseline data for the area. Species Conservation efforts should thus target water bodies of varying habitat types to protect greater species diversity.

## Introduction

Freshwater ecosystems contain 10% of current recorded species and comprise only 1% of Earth's surface [1]. They are considered one of the most jeopardized ecosystems [2] and their importance as a resource in undeniable. To better monitor the state and health of these ecosystems, indicator species are often used. Odonata (dragonflies) make particularly good indicators of freshwater ecosystem health as they are visible above water, but rely on the quality of the water and surrounding habitat to persist [3], [4]. Among insects Odonata have comparatively long life cycles and as a group are well defined and studied [3]–[5]. They have an aquatic larval stage that can last up to one year and a terrestrial adult phase, with males holding favourable territories in many species [6]. Consequently, they serve as indicators for changes in both water quality and surrounding vegetation [7], [8]. Their value as flagship species for freshwater conservation is further highlighted by their important role within freshwater ecosystem species assemblages and their presence on all continents, with the exception of Antarctica [3], [4]. Odonata assemblages can also be used as surrogates to determine aquatic areas for conservation prioritization [9].

iSimangaliso Wetland Park (iWP), South Africa, is known for rich diversity and unique habitats and is therefore a Ramsar wetland of global significance and a UNESCO World Heritage Site. It is located within Maputaland at a significant intersection, with the coastal lowlands bordered by the ocean to the east and an inland plateau to the west [10]. Maputaland's position lends itself to colonization by tropical biota from the north and sub-tropical and temperate biota from both the south and high altitude west [10]. Being a transition zone between these environments has resulted in great biodiversity [10]. Maputaland's conservation value as a centre of endemism is internationally recognised [11]. Today it is accepted that predominantly tropical species are found in the area, largely due to warm ocean currents flowing south from Mozambique, presenting a rich and diverse ecosystem at relatively high latitude [10]. It is unique as it is made up of several habitat types including estuaries, coastal/marine habitats, freshwater lakes and rivers, wetlands, dune and coastal and swamp forests, and mangroves. Many of the vegetation units are vulnerable or endangered outside of the protected iWP, where agricultural practices and invasive alien plants pose the biggest threats [12]. Africa's largest estuarine system, Lake St. Lucia, and southern Africa's largest natural freshwater lake, Lake Sibaya, are both found within iWP [13], [14].

Within South Africa, Maputaland–Pondoland–Albany (MPA) has been identified as a hotspot with the greatest Odonata richness, particularly for red-listed species [9]. The iWP's diverse odonate fauna is due to the subtropical climatic conditions with relatively high rainfall, and variable landscapes and wetland types within the park. Odonata assemblages are associated with different habitat types [15]. Consequently, increased habitat heterogeneity can lead to increased Odonata diversity at a particular site [16], [17]. Of South Africa's 162 taxa, one quarter are Red Listed [9]. The greatest threats to Odonata are those that alter the natural landscape [17]. These include: invasive tree species which cause excessive shading, urbanization, pollution, damming, mining, and introduced fish species [9], [17]–[19] to name a few.

Disturbance to these habitats can result in a reduction of odonate species [7]. Odonata assemblages should therefore be monitored to recognize what effect human actions have on water quality [20]. Therefore species lists for wetland areas are important as these will serve as baseline data and may indicate changes within the ecosystem. Furthermore, information on hotspots within a reserve can serve as focal points for management to direct cost-effective conservation strategies [21]. Finally, it is important that all habitat types be surveyed within an area as these can yield different species assemblages.

As the MPA is a biodiversity hotspot and centre for endemism, and Odonata are indicators of freshwater ecosystem health, the aim of this study was to determine the Odonata diversity of the iWP. In addition we compared the odonate species composition at different sites to illustrate habitat importance. From this odonate data we detailed important species in terms of endemism, conservation status, and potential as indicator species. It was predicted that odonate species assemblages would differ at sites that varied in habitat type, and so affect conservation management strategies.

## Materials and Methods

### Study area

**Figure 1:**
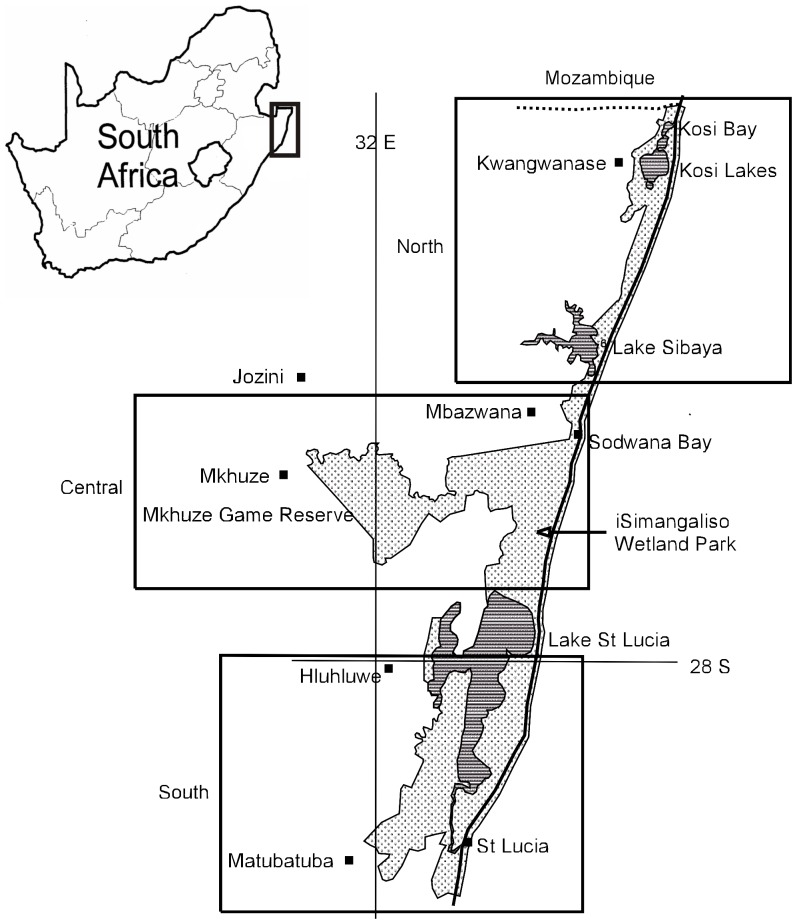
Map of study area. Grey shaded areas indicate the iSimangaliso Wetland Park.

The iWP (26°51′S–28°26′S; 32°09′E–32°53′E) extends along the coastal plain of north-eastern KwaZulu-Natal (KZN) Province of South Africa and covers an area of 332000 ha. It stretches from Maphelana in the south to the Mozambique border, north of Kosi Bay, in the north and extends inland for approximately 50 km to include Mkhuze Game Reserve (Fig 1). In general most of the park is less than 40 m above sea-level, with the exception of Mkhuze Game Reserve (c. 60–100 m). Rainfall varies greatly, with the coastal area receiving 1000–1100 mm annually and decreasing to 600 mm in the west at the foot of the mountain range [22].

**Figure 2:**
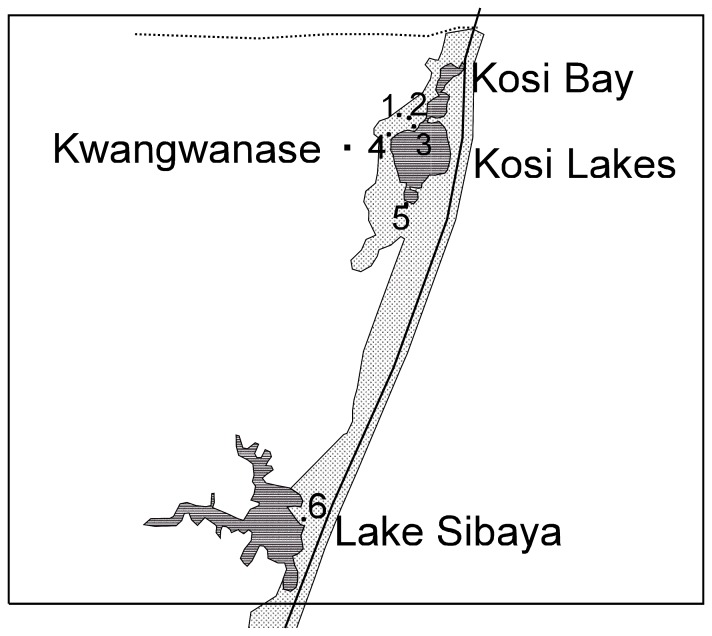
Map of the northern section of the iSimangaliso Wetland Park. The numbers 1–6 represent the positions of sample sites described in Table 1.

**Figure 3:**
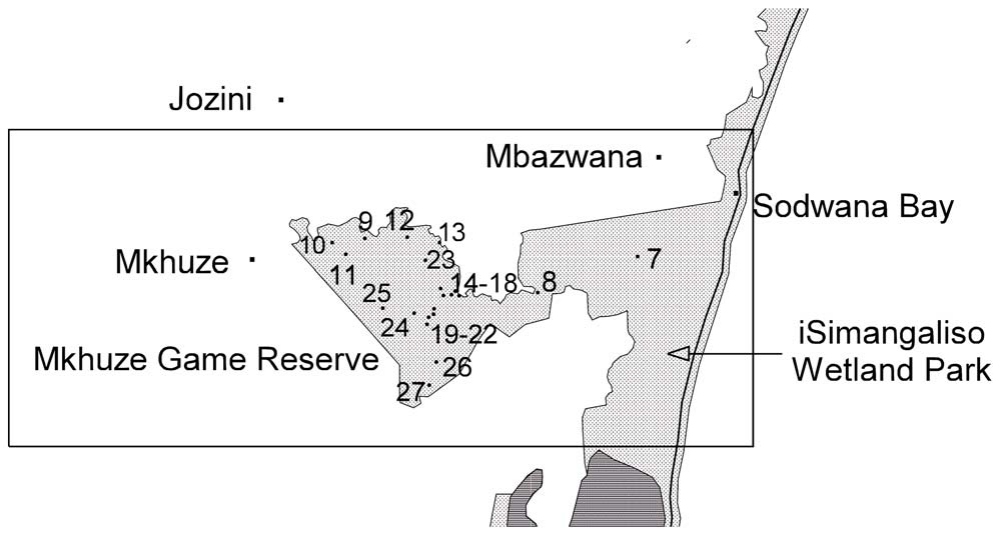
Map of the central section of the iSimangaliso Wetland Park. The numbers 7–27 represent the positions of sample sites described in Table 1.

**Figure 4:**
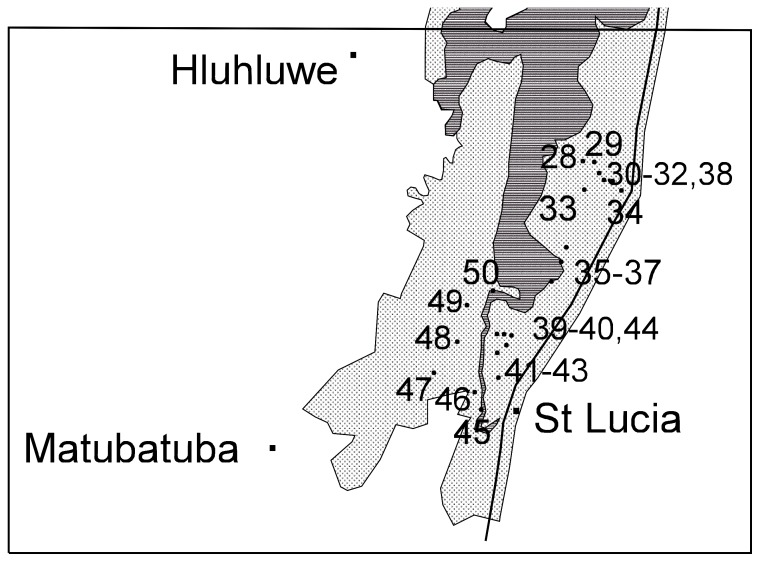
Map of the southern section of the iSimangaliso Wetland Park. The numbers 28–50 represent the positions of sample sites described in Table 1.

We had permission from the local conservation authority for the odonate species surveys. The iWP was divided into three sections; north (Fig 2), central (Fig 3) and south (Fig 4). Within these sections a total of fifty sites were identified and numbered accordingly (Table 1; Fig. 2, 3, 4). For brevity, site names were abbreviated from Kosi Bay to Kosi, Mkhuze Game Reserve to Mkhuze, etc. Most sites were photographed and a description of each site was provided (Table 1). GPS co-ordinates, air temperature, and the total dissolved solids (TDS) of the water were also recorded for each site (Table 1). TDS was measured using Milwaukee Instruments CD97 Total Dissolved Salts (TDS) meter.

**Table 1:** Description of study sites in each area surveyed.

Area	Site No.	Site name	Site Description	Latitude	Longitude	Temp. (°C)	Humidity (%)	TDS (ppm)	No. of Species
Kosi	1	Kosi	10 ha rain-filled grassy pan in grassland.	26.952050	32.802417	35.3	41	56	12
Kosi	2	Kosi	Forested stream.	26.957267	32.829883			104	14
Kosi	3	Kosi campsite & jetty	Edge of lake.	26.960200	32.826967			650	7
Kosi	4	Kosi	Swamp forest stream.	26.955033	32.828833	31.2	40	101	4
Kosi	5	Inlet to 4th Lake	Narrow inlet, *Nymphaea*.	27.040300	32.818917			97	20
Sibaya	6	Sibaya	Eastern shore	27.395650	32.711633	30.5	58	95	7
Ozabeni	7	Samango Crossing	Flowing stream in forest	27.617467	32.549183				12
Ozabeni	8	Neshe Pan	Open water, *Nymphaea*.	27.654700	32.402650	28.1	58	221	14
Mkhuze	9	Nhlonhlela Bush Camp	Floodplain below a nearly dry reedbed	27.597450	32.198167			97	3
Mkhuze	10	Rhino wallow	Small 50×10 m, shallow, seasonally rain-filled wallow with emergent grass. Disturbed by game.	27.607817	32.167400			210	4
Mkhuze	11	Rhino wallow	Small 50×10 m, shallow, seasonally rain-filled wallow with emergent grass. Disturbed by game.	27.621733	32.185733			120	2
Mkhuze	12	Mbonene Pan	Small, rain-filled, grass edge.	27.632817	32.262767	30	54	53	7
Mkhuze	13	Ophansi bridge	Mkuze river bridge, fast-flowing, mud-laden River 20 m wide, fringed by degraded fig forest.	27.598750	32.302083	24/25.9*	75*	297/255*	5/10*
Mkhuze	14	Nsumo Pan	Bridge at first inlet 3 m wide open water channel fringed by flooded grass.	27.656600	32.301417	21/32*	49*	405/175*	3/12*
Mkhuze	15	Nsumo Pan	West hide open water, with reeds along edge	27.665117	32.302150	33.5	51	139	7/2*
Mkhuze	16	Nsumo Pan Picnic site	Open water, with reeds along edge.	27.668750	32.305400			324	5/5*
Mkhuze	17	Fig Forest	First bridge 10 m wide open water channel fringed by flooded grass and thick bush.	27.668783	32.316717				21/13*
Mkhuze	18	Fig Forest	Second bridge strongly flowing, mud-laden river fringed by tall fig forest and shrubby understory.	27.669517	32.322850			340/231*	8/9*
Mkhuze	19	near Nxwala Camp	Shaded, stagnant residual pond on seasonal drainage line in dense bush.	27.703150	32.284333	32.1/24*	48	96/144*	11/5*
Mkhuze	20	Nsumo Nxwala side	Lily-covered channel backfill from Nsumu Pan. *Nymphaea* covered water below fever trees.	27.692050	32.291050			155	12/7*
Mkhuze	21	Nsumo Nxwala side	Nsumo western inlet, flooded grass below fever trees	27.686550	32.292433			363	8
Mkhuze	22	Nsumo Nxwala side	Nsumo western inlet, flooded grass below fever trees	27.689783	32.293600	23			7/12*
Mkhuze	23	Ediza	Inlet, dense flooded grass	27.606617	32.288250	31.8	44		4
Mkhuze	24	Rhino wallow	Small 50×10 m, shallow, seasonally rain-filled wallow with emergent grass. Disturbed by game.	27.692517	32.278633			91	7
Mkhuze	25	Mkhuze	Rain-filled quarry, open water with flooded grass edges.	27.685833	32.238167	27			4
Mkhuze	26	Noshoshela dam	Dam of 2 ha, open water fringed by fever trees.	27.756983	32.297300	32.3	43	98/140*	10/22*
Mkhuze	27	uMkhumbe dam	Dam of 1 ha, open water fringed by extensive flooded grass with narrow channel of flowing water below the dam.	27.775117	32.297800	28		169/98*	18/13*
E Shores	28	E Shores	Grassy pan.	28.118083	32.506783	29.7	57	225	11
E Shores	29	E Shores	Small pan.	28.118417	32.515467	28.2	58		3
E Shores	30	E Shores	Forested causeway and Mfabeni Swamp.	28.131117	32.527167	34.8	47	345	8
E Shores	31	E Shores	Forested causeway and Mfabeni Swamp.	28.137000	32.534683	32.8	61	280	10
E Shores	32	E Shores	Lake Bangazi road.	28.141367	32.541100	24	68		6
E Shores	33	E Shores	Barbet Pan.	28.194633	32.489200	28	60	225	8
E Shores	34	Cape Vidal house	Garden and road.	28.146333	32.547933	32	55		3
E Shores	35	E Shores	Forested stream and old excavations.	28.205900	32.490700				12
E Shores	36	Catalina jetty	Freshwater edge on Lake St Lucia.	28.220650	32.487283	32.3	58	174	8
E Shores	37	E Shores	Freshwater seep on Lake St Lucia edge.	28.238867	32.486950			Seep 63, Lake 974	20
E Shores	38	E Shores	Forested causeway and Mfabeni Swamp.	28.137550	32.538567				7
E Shores	39	E Shores	Dense flooded sedge beds.	28.296617	32.434383				10
E Shores	40	E Shores	Freshwater seep on swamp-forest edge.	28.297150	32.440900	27	59	85	13
E Shores	41	E Shores	Two rhino, open grassy pans.	28.316500	32.433717	26.9	67	85	13
E Shores	42	E Shores	Papyrus choked pond.	28.318550	32.427050	31.8	64	129	3
E Shores	43	E Shores	Two grassy pans divided by causeway.	28.318583	32.430400	32.5	61	88	10
E Shores	44	E Shores	Warthog grassy pan.	28.268267	32.466783	33.9	53	110	3
W Shores	45	W Shores	St Lucia estuary bridge, reedbed.	28.369783	32.409667	31	63		7
W Shores	46	Ndonyena	Small lily pond.	28.352850	32.385350	31.6	57	101	3
W Shores	47	Mpati Weir	Flowing stream under forest canopy	28.331200	32.361367	32.4	54	97	5
W Shores	48	Mpati River	Stream low, not flowing, choked with Phragmites	28.298467	32.383600	34.4	51	536	10
W Shores	49	W Shores	Hippo pan, open grassy.	28.255017	32.393750	34.1	50	108	8
W Shores	50	Makakatana Bay	Saline open water, bare edges.	28.248900	32.419617	33.5	46	940	4

Site numbers refer to site positions marked on Fig 1. ‘*’ indicates a second reading or count for the same site.

### Odonata identification and analyses

A checklist for possible species in the area was compiled using the database compiled by Ezemvelo KwaZulu-Natal Wildlife (EKZNW), which listed 486 records of 70 odonate species for the iWP (Table 2). Nearly 52% of these records were collected from 1997–2001 and are accredited to Samways and the University of KwaZulu-Natal. Records and a dragonfly collection for St. Lucia and elsewhere in the iWP held at the National Museum in Pretoria (Ditsong) from Balinsky [23], [24] were also consulted. Finally, three species were added from literature searches.

**Table 2:** Species presence in each region and the number of sites at which they were recorded.

Family	Species	Common name	Samango Crossing	Mkhuze	Neshe Pan	E & W Shores	Lake Sibaya	Kosi Bay	EKZNW database	Other records	No. of our sites
Calopterygidae	*Phaon iridipennis*	Glistening Demoiselle		yes				yes	yes		4
Chlorocyphidae	*Platycypha caligata*	Dancing Jewel	yes	yes				yes	yes		6
Coenagrionidae	*Aciagrion dondoense*	Opal Slim								yes	
Coenagrionidae	*Africallagma glaucum*	Swamp Bluet							yes	yes	
Coenagrionidae	*Agriocnemis exilis*	Little Whisp				yes		yes	yes		3
Coenagrionidae	*Agriocnemis falcifera*	White-masked Whisp							yes	yes	
Coenagrionidae	*Agriocnemis gratiosa*	Gracious Whisp							yes		
Coenagrionidae	*Agriocnemis ruberrima*	Orange Whisp				yes		yes	yes		2
Coenagrionidae	*Azuragrion nigridorsum*	Black-tailed Bluet		yes		yes		yes	yes		5
Coenagrionidae	*Ceriagrion glabrum*	Common Citril		yes	yes	yes		yes	yes		36
Coenagrionidae	*Ischnura senegalensis*	African Bluetail		yes	yes	yes		yes	yes		16
Coenagrionidae	*Pseudagrion acaciae*	Acacia Sprite		yes					yes		1
Coenagrionidae	*Pseudagrion coeleste*	Umsingazi Sprite		yes	yes			yes			9
Coenagrionidae	*Pseudagrion commoniae*	Black Sprite		yes					yes		3
Coenagrionidae	*Pseudagrion hageni*	Hagen's Sprite	yes			yes		yes	yes		5
Coenagrionidae	*Pseudagrion hamoni*	Hamon's Sprite		yes				yes	yes		8
Coenagrionidae	*Pseudagrion kersteni*	Kersten's Sprite		yes				yes			3
Coenagrionidae	*Pseudagrion massaicum*	Masai Sprite		yes	yes			yes	yes		17
Coenagrionidae	*Pseudagrion sublacteum*	Cherry-eye Sprite		yes					yes		4
Platycnemididae	*Elattoneura glauca*	Common Threadtail	yes					yes	yes		3
Lestidae	*Lestes pallidus*	Pallid Spreadwing		yes							4
Lestidae	*Lestes tridens*	Spotted Spreadwing		yes				yes	yes		2
Lestidae	*Lestes uncifer*	Sickle Spreadwing							yes		
Gomphidae	*Ictinogomphus ferox*	Common Tigertail	yes	yes	yes		yes	yes	yes		14
Gomphidae	*Paragomphus cognatus*	Rock Hooktail	yes					yes			2
Gomphidae	*Paragomphus genei*	Green Hooktail				yes	yes	yes	yes		3
Aeshnidae	*Zosteraeschna minuscula*	Friendly Hawker								yes	
Aeshnidae	*Anaciaeschna triangulifera*	Evening Hawker						yes	yes		1
Aeshnidae	*Anax ephippiger*	Vagrant Emperor	yes	yes		yes			yes		9
Aeshnidae	*Anax imperator*	Blue Emperor		yes		yes	yes	yes	yes		10
Aeshnidae	*Anax speratus*	Orange Emperor						yes			1
Aeshnidae	*Anax tristis*	Black Emperor								yes	
Aeshnidae	*Gynacantha manderica*	Little Dusk-Hawker		yes							2
Aeshnidae	*Gynacantha usambarica*	Usambara Dusk-Hawker						yes	yes		2
Aeshnidae	*Gynacantha villosa*	Hairy Dusk-Hawker							yes		
Corduliidae	*Hemicordulia africana*	African Emerald						yes	yes		1
Corduliidae	*Phyllomacromia contumax*	Two-banded Cruiser		yes			yes	yes	yes		3
Corduliidae	*Phyllomacromia picta*	Darting Cruiser		yes							2
Libellulidae	*Acisoma panorpoides*	Pintail		yes		yes		yes	yes		10
Libellulidae	*Aethriamanta rezia*	Pygmy Basker				yes		yes	yes		2
Libellulidae	*Brachythemis leucosticta*	Banded Groundling		yes	yes	yes		yes	yes		34
Libellulidae	*Bradinopyga cornuta*	Don-Dwala								yes	1
Libellulidae	*Chalcostephia flavifrons*	Inspector		yes		yes		yes	yes		8
Libellulidae	*Crocothemis erythraea*	Broad Scarlet		yes	yes	yes		yes	yes		34
Libellulidae	*Crocothemis sanguinolenta*	Little Scarlet							yes		
Libellulidae	*Diplacodes lefebvrii*	Black Percher		yes		yes		yes	yes		19
Libellulidae	*Diplacodes luminans*	Barbet		yes		yes	yes	yes	yes		14
Libellulidae	*Diplacodes pumila*	Dwarf Percher				yes			yes		1
Libellulidae	*Hemistigma al bipunctum*	Pied-Spot		yes		yes		yes	yes		23
Libellulidae	*Macrodiplax cora*	Cora's Pennant							yes	yes	
Libellulidae	*Nesciothemis farinosa*	Black-tailed Skimmer	yes	yes	yes	yes	yes	yes	yes		20
Libellulidae	*Notiothemis jonesi*	Forest-Watcher		yes							1
Libellulidae	*Orthetrum abbotti*	Abbott's Skimmer				yes			yes		2
Libellulidae	*Orthetrum chrysostigma*	Epaulet Skimmer							yes		
Libellulidae	*Orthetrum hintzi*	Hintz's Skimmer	yes			yes			yes		3
Libellulidae	*Orthetrum icteromelas*	Spectacled Skimmer				yes		yes	yes		4
Libellulidae	*Orthetrum julia*	Julia Skimmer	yes	yes		yes		yes	yes		15
Libellulidae	*Orthetrum machadoi*	Machado's Skimmer							yes		
Libellulidae	*Orthetrum robustum*	Robust Skimmer				yes		yes	yes		4
Libellulidae	*Orthetrum stemmale*	Strong Skimmer		yes		yes			yes		7
Libellulidae	*Orthetrum trinacria*	Long Skimmer		yes	yes	yes	yes	yes	yes		17
Libellulidae	*Palpopleura jucunda*	Yellow-veined Widow				yes					1
Libellulidae	*Palpopleura lucia*	Lucia Widow		yes		yes		yes	yes		16
Libellulidae	*Palpopleura portia*	Portia Widow				yes					2
Libellulidae	*Pantala flavescens*	Pantala	yes	yes		yes		yes	yes		27
Libellulidae	*Parazyxomma flavicans*	Banded Dusk-Darter		yes							2
Libellulidae	*Rhyothemis semihyalina*	Phantom Flutterer		yes	yes	yes		yes	yes		20
Libellulidae	*Sympetrum fonscolombii*	Nomad							yes		
Libellulidae	*Tetrathemis polleni*	Black-Splash		yes		yes		yes	yes		9
Libellulidae	*Tholymis tillarga*	Twister				yes			yes		2
Libellulidae	*Tramea basilaris*	Keyhole Glider	yes	yes		yes		yes	yes		28
Libellulidae	*Tramea limbata*	Ferruginous Glider				yes			yes		2
Libellulidae	*Trithemis aconita*	Monkshood Dropwing						yes			1
Libellulidae	*Trithemis annulata*	Violet Dropwing		yes	yes	yes		yes	yes		15
Libellulidae	*Trithemis arteriosa*	Red-veined Dropwing		yes	yes	yes		yes	yes		16
Libellulidae	*Trithemis dorsalis*	Dorsal Dropwing							yes		
Libellulidae	*Trithemis furva*	Navy Dropwing	yes						yes		1
Libellulidae	*Trithemis hecate*	Hecate Dropwing						yes	yes		1
Libellulidae	*Trithemis kirbyi*	Kirby's Dropwing		yes					yes		3
Libellulidae	*Trithemis pluvialis*	River Dropwing							yes		
Libellulidae	*Trithemis stictica*	Jaunty Dropwing						yes	yes	yes	2
Libellulidae	*Urothemis assignata*	Red Basker		yes	yes	yes		yes	yes		14
Libellulidae	*Urothemis edwardsii*	Blue Basker		yes	yes	yes		yes	yes		9
Libellulidae	*Urothemis luciana*	St Lucia Basker							yes		
Libellulidae	*Zygonyx torridus*	Ringed Cascader							yes		
Libellulidae	*Zyxomma atlanticum*	Little Dusk-Darter						yes	yes		1
											
		Total species recorded	12	43	14	39	7	49	70	8	-
		Total number counted	86	799	150	1437	16	288	-	-	-
	Dragonfly Biotic Index/Site		1.67	2.05	1.57	2.38	1.86	2.59	-	-	-
	Dragonfly Biotic Index		20	88	22	93	13	127			

Previous records from other sources are also presented.

Species names are based on Samways [27], where full names including authors are given.

At each site odonate species were identified and counted. Identification of species was predominantly done using close-focusing binoculars. In many instances at least one individual of each species was caught and examined using a hand-lens to confirm identification and subsequently released. In addition most species were also photographed to provide a permanent record of identification and occurrence. In addition to formal surveys incidental observations were also recorded. Odonata were surveyed in Mkhuze Game Reserve and surrounds for six days in December 2009, and Eastern and Western Shores of Lake St. Lucia, Mkhuze Game Reserve, Lake Sibaya and Kosi Bay were covered over a 10 day period in February 2011. Identifications were made using the two field-guides of Tarboton and Tarboton [25], [26] and Samways [27], and from literature extracts accumulated by Tarboton.

A map of the study sites was created using ESRI ArcView GIS version 3.1. A detailed species list was compiled for iWP. Using this list, the Dragonfly Biotic Index (DBI) for each study area was determined. The DBI assigns a value ranging from 0–9 to each odonate species in South Africa [27]. This value incorporates the geographical distribution, conservation status and sensitivity to habitat change of a species, where a species scoring ‘0’ would be widespread, common and tolerant to human disturbance [27], [28]. To determine the DBI/site, the total DBI for each study area was divided by the number of species recorded at each of these and thus yielded a DBI/site value between 0–9 for each area [29], [30]. To test which study areas were most similar in species composition Non-metric Multidimensional Scaling (NMDS) was run with a Jaccard similarity coefficient (Primer E, ver. 6, UK). For Mkhuze and Kosi Bay, where there was more than one sampling trip, species composition was totalled.

## Results

In total 68 species and 3734 individual Odonata were recorded at the study sites. The summation of these data provides evidence for 86 species of odonates occurring in the iWP. From the compiled checklist, two species that are recorded in the EKZNW database were rejected based on our observations. Surveys from this study provide an additional 13 species to the iWP checklist. Based on results from this study, the EKZNW database, and published records [23], [24], [27], [31]–[33] an annotated checklist for the iWP has been compiled and the DBI for each study area calculated (Table 2). An indication of relative abundance and known occurrence of each species in the iWP is provided in Appendix S1. Family and species nomenclature are revised to the currently accepted position as listed in Samways [27]. Species showed a range of DBI scores, ranging from 0–8. Based on the checklist of 86 species for the iWP the total possible DBI/site is 2.80 (Total DBI = 241; Table 2). In this study 68 species were observed with a total DBI/site of 2.57 (Total DBI = 175; Table 2). When considering the six study areas, the highest DBI/site of 2.59 was at Kosi Bay, while the lowest value of 1.57 was at Neshe Pan (Table 2).

Of the species identified, eight appear in the National Red List of South African Odonata [33], namely: *Aciagrion dondoense*, *Agriocnemis gratiosa*, *Agriocnemis ruberrima* subspecies *ruberrima*, *Pseudagrion coeleste* subspecies *umsingaziense*, *Gynacantha villosa*, *Diplacodes pumila* and *Urothemis luciana*. These species ranges extend north, into Mozambique, with some widely ranging into tropical Africa. Within South Africa, 12 of the identified Odonata have restricted distributions in the coastal plains of northern KwaZulu-Natal. The remaining species occur broadly across the southern African savanna. The 10 most abundant species from this study are largely similar to those in the EKZNW database. These include: B*rachythemis leucosticta* 920/24/25 (our count/records in EKZNW database/sites present); *Hemistigma albipunctum* 280/19/21; *Pantala flavescens* 276/8/26; *Crocothemis erythraea* 204/12/28; *Ceriagrion glabrum* 190/25/31; *Diplacodes luminans* 147/4/12; *Tramea basilaris* 143/12/27; *Diplacodes lefebvrii* 114/22/18; *Palpopleura lucia* 126/20/12; and *Ischnura senegalensis* 107/16/16. *Ceriagrion glabrum*, *Crocothemis erythraea* and *Tramea basilaris* were present at the most sites surveyed. Two doubtful species we suggest be removed from the checklist are *Phyllogomphus brunneus* and *Ceriagrion suave*. Reasons for this are discussed in Appendix S1.

**Figure 5:**
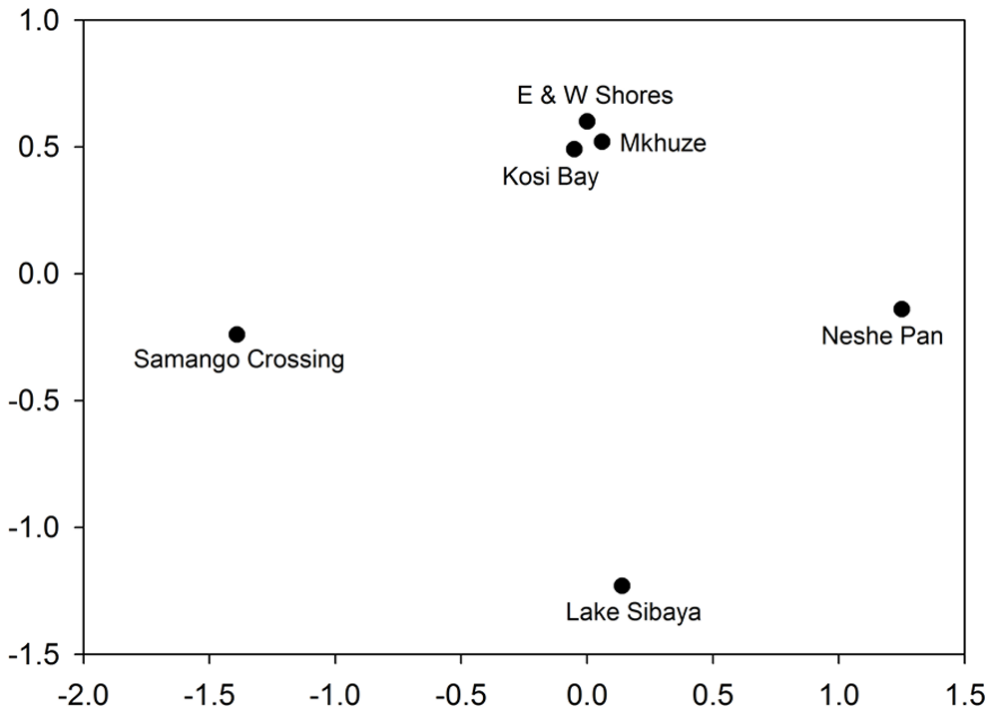
Study areas' similarity in species composition. Multidimensional scaling of study areas namely: Kosi Bay, Eastern and Western Shores, Mkuze, Neshe Pan, Samango Crossing, and Lake Sibaya, based on their Jaccard index similarity matrices using presence/absence data for all Odonata species observed.

The NMDS plot illustrating the similarity in Odonata species composition between sites, showed that Kosi Bay, Eastern and Western Shores, and Mkhuze were most similar (Fig. 5). Samango Crossing, Neshe Pan and Lake Sibaya were least similar in composition to any of the other study sites (Fig. 5).

## Discussion

Based on data from both the formal surveys and incidental observations, 68 odonate species were observed in this study in iWP. Thirteen species not previously recorded for this park were identified. To date 86 species have now been recorded for iWP. This total is just over 50% of the total recorded for South Africa [27]. It also exceeds Kruger National Park (n = 81), an area approximately six fold larger [34], [35]. In Africa more than 80% of odonate species, and over 70% of globally threatened species, occur within protected areas, which are largely fragmented and isolated [36]. The greater Odonata diversity at iWP is largely due to the diverse habitat types present; in particular coastal swamp forest (with *Barringtonia*), which supports several elusive odonates (e.g. *Gynacantha, Hemicordulia* and others) that do not range inland. The remaining odonate diversity generally resembles assemblages typical of the (southern) African savanna. Some common savanna species (e.g. *Africallagma glaucum, Sympetrum fonscolombii, Pseudagrion kersteni, Paragomphus cognatus, Trithemis kirbyi*) are however rare, or absent from the park. This could be due to climatic or other factors, for example pH, which has been shown to be strongly correlated with dragonfly diversity [18] and prevents their range from extending to the coast. Species which favour lentic wetlands dominate iWP assemblages and savanna species dependent on lotic wetlands, especially perennial streams and rivers, are least represented. This reflects the paucity of such biotopes in the park.

DBI's can be used to identify areas of conservation importance [9]. The DBI of the area under which iWP falls, has previously been identified as relatively high and therefore of conservation significance [37]. DBI's provide a useful tool for monitoring changes in odonate assemblages, for example those resulting from invasive alien plant disturbances [28], [38], [39] or changes due to human alteration of ecosystems [40]. The total DBI's observed for Kosi Bay, Eastern and Western Shores, and Mkhuze were considerably higher than values for sites in the Tsitsikamma region, Western and Eastern Cape Provinces in South Africa, although DBI/site values were lower [41]. Higher DBI/site scores can be explained by the presence of fewer, rare species and therefore higher individual DBI scores in an area. Although study areas in iWP had high species numbers, most DBI scores for species were four or less.

Of the Red Listed odonate species the iWP likely plays a significant conservation role for Urothemis luciana and Pseudagrion coeleste subspecies. Gynacantha villosa and usambarica, Hemicordulia africana, Aethriamantra rezia, Chalcostephia flavifrons and Macrodiplax cora, are also species of local interest as their South African ranges are confined to coastal Zululand. The records of Macrodiplax cora in iWP warrant further investigation, as these are the only known occurrences in Africa south of Somalia, of this fundamentally Asian species.

Many sections of iWP remain to be surveyed as some odonate species listed are based on a single known occurrence. Further surveys are required as we believe that *Platycypha fitzsimonsi, Lestes plagiatus, Lestes virgatus, Pseudagrion gamblesi, Pseudagrion salisburyense, Pseudagrion sudanicum, Lestinogomphus angustus, Crenigomphus hartmanni, Ceratogomphus pictus, Orthetrum caffrum, Orthetrum guineense, Palpopleura deceptor, Brachythemis lacustris, Trithemis donaldsoni* and *Zygonoides fuelleborni* could be present in this region. Additionally, sites surveyed in Mkhuze indicated seasonal variability (e.g. in numbers of *Phaon iridipennis*, *Gynacantha manderica* and *Brachythemis leucosticta*). The EKZNW database and published records also indicate that several species temporarily extend their ranges into this area from the tropics during high rainfall years. This is not uncommon for these vagile organisms and is a trait which also contributes to their re-colonization of recovering habitats [42]. Such events would contribute to additional Odonata species. Finally, it is also important to cover water bodies of varying sizes as these can also yield different species assemblages [43].

Species composition for Eastern and Western Shores, Mkhuze and Kosi Bay shows a strong similarity. These three zones all include a range of habitat types, including permanent and temporary pans, flowing water, riverine vegetation and some forest. Kosi Bay and Eastern and Western Shores are both on the coastal plain and share a very similar geography.

Species composition for Neshe Pan was dissimilar to the other sites even though geographically it is close (12 km) to the Mkhuze sites. Neshe Pan is very different from the other pans that were sampled. It is a temporary pan on the Mkuze River, is not tree-lined, and is outside of any conservation area. In dry periods the area is cultivated, and these lands are then flooded when the river flows strongly. The vegetation of Neshe Pan is particularly suitable for odonate breeding and survival. The pan is shallow and has thick beds of reeds and large areas of *Nymphaea*, offering ideal breeding and feeding habitats for the larval stages. It also offers large feeding areas and many territories for adults.

Samango Crossing odonate species composition was least similar to the other water bodies surveyed in this study. This can be explained by the unique habitat there. It has a greater variation of habitat types within a small area when compared to the other sites. It has the typical vegetation of the coastal plain, but also has the fresh water Manzibomvu stream flowing through it. The stream flows beneath a canopy of swamp forest trees and there are inlets of stationary water.

The species composition for Lake Sibaya is also dissimilar to all other sites. This can be ascribed to the nature of this lake. It is positioned just behind the first dune, and is a large, clear lentic system. It is lined with dune forest on its eastern edge, and supports very little aquatic vegetation or reed beds.

Based on results from this study, it is clear that within South Africa in particular, iWP is an important area for the conservation of Odonate diversity. This is largely due to the diverse habitats found within iWP and the potential to be colonized by both tropical and temperate species [10]. Furthermore, iWP is a protected area thereby reducing the direct negative impacts to its water bodies and benefiting from monitoring and management practices. As a population, Odonata fulfil many ecosystem services either directly or indirectly [26]. These are broadly grouped into: provisioning, cultural, supporting, and regulating services [26]. Odonata vary in their sensitivity to environmental change, and while some individual species can indicate change (e.g. [25]); it is recommended that changes in odonate assemblages as a whole be considered as indicators of environmental disturbance [26]. Thus surveys of Odonata diversity, particularly within ecologically important areas such as iWP, are invaluable.

Odonata respond to climatic and environmental changes [42]. In light of global climate change understanding shifts in species assemblages and the associated implications of such changes becomes increasingly important. Logistic constraints highlight the need for an indicator species group to facilitate rapid and continued surveys in a changing environment [30], [44]. The traits of Odonata lend them to fulfill this essential role [7], [8], [30]. Maputaland was recognized for its unique habitat and as an area of significant biodiversity, thereby motivating for the establishment of a large protected area, today known as iWP [45]. The diverse habitat types within the iWP support a great diversity of Odonata, reiterating its role particularly in the conservation of aquatic diversity.

## Supporting Information

Appendix S1 Odonata species account for iSimangaliso Wetland Park. Nine families are arranged in taxonomic order, with species accounts appearing alphabetically.
(DOC)

## References

[1] Strayer DL, Dudgeon D (2010) Freshwater biodiversity conservation: recent progress and future challenges. Journal of the North American Benthological Society 29: 344–358.

[2] Dudgeon D, Arthington AH, Gessner MO, Kawabata ZI, Knowler DJ, et al (2006) Freshwater biodiversity: importance, threats, status and conservation challenges. Biological Reviews 81: 163–182. 16336747 10.1017/S1464793105006950

[3] Clausnitzer V, Kalkman VJ, Dijkstra K-DB, Ram M, Collen B, et al (2009) Odonata enter the biodiversity crisis debate: the first global assessment of an insect group. Biological Conservation 142: 1864–1869.

[4] Kalkman VJ, Clausnitzer V, Dijkstra K-DB, Orr AG, Paulson DR, et al (2008) Global diversity of dragonflies (Odonata) in freshwater. Hydrobiologia 363: 595–351.

[5] Wissinger SA (1988) Life history and size structure of larval dragonfly populations. Journal of the North American Benthological Society 7: 13–28.

[6] Picker M, Griffiths C, Weaving A (2002) Field Guide to Insects of South Africa. Cape Town: Struik Publishers.

[7] Dolny A, Bárta D, Lhota S, Rusdianto, Drozd P (2011) Dragonflies (Odonata) in the Bornean rain forest as indicators of changes in biodiversity resulting from forest modification and destruction. Tropical Zoology 24: 63–86.

[8] Stewart DAB, Samways MJ (1998) Conserving dragonfly (Odonata) assemblages relative to river dynamics in an African savanna game reserve. Conservation Biology 12: 683–692.

[9] Simaika JP, Samways MJ (2009) Reserve selection using Red Listed taxa in three global biodiversity hotspots: Dragonflies in South Africa. Biological Conservation 142: 638–661.

[10] Bruton MN (1980a) Introduction. In: Bruton MN, Cooper KH, editors. Studies on the Ecology of Maputaland: Rhodes University and Natal Branch of the Wildlife Society of Southern Africa, Durban. pp. xvii-xix.

[11] Steenkamp Y, Van Wyk B, Victor J, Hoare D, Smith G, et al (2004) Maputaland–Pondoland–Albany. In: Mittermeier RA, Robles-Gil P, Hoffmann M, Pilgrim J, Brooks T, et al., editors. Hotspots Revisited: Earth's Biologically Richest and Most Endangered Ecoregions. Mexico City, Mexico: Cemex. pp. 219–228.

[12] Mucina L, Scott-Shaw CR, Rutherford MC, Camp KGT, Matthews WS, et al. (2006) Indian Ocean Coastal Belt. In: Mucina L, Rutherford MC, editors. The vegetation of South Africa, Lesotho and Swaziland South African National Biodiversity Institute, Pretoria. pp. 584–615.

[13] Humphries MS (2013) DDT residue contamination in sediments from Lake Sibaya in northern KwaZulu-Natal, South Africa: Implications for conservation in a World Heritage Site. Chemosphere 93: 1494–1499. 23972730 10.1016/j.chemosphere.2013.07.047

[14] Taylor RH (1995) St-Lucia Wetland Park. Cape Town, South Africa: Struik Publishers.

[15] Bried JT, Ervin GN (2005) Distribution of adult Odonata among localized wetlands in east-central Mississippi. Southeastern Naturalist 4: 731–744.

[16] Dolny A, Harabiš F (2012) Underground mining can contribute to freshwater biodiversity conservation: Allogenic succession forms suitable habitats for dragonflies. Biological Conservation 145: 109–117.

[17] Clausnitzer V, Dijkstra K-DB, Koch R, Boudot J-P, Darwall WRT, et al (2012) Focus on African freshwaters: hotspots of dragonfly diversity and conservation concern. Frontiers in Ecology and the Environment 10: 129–134.

[18] Kinvig R, Samways MJ (2000) Conserving dragonflies (Odonata) along streams running through commercial forestry. Odonatologica 29: 195–208.

[19] Samways MJ, Taylor S (2004) Impacts of invasive alien plants on Red-Listed South African dragonflies (Odonata). South African Journal of Science 100: 78–80.

[20] Suhling F, Sahlen G, Martens A, Marais E, Schutte C (2006) Dragonfly assemblages in arid tropical environments: a case study from western Namibia. Biodiversity and Conservation 15: 311–332.

[21] Grant PBC, Samways MJ (2011) Micro-hotspot determination and buffer zone value for Odonata in a globally significant biosphere reserve. Biological Conservation 144: 772–781.

[22] Maud RR (1980) The climate and geology of Maputaland. In: Bruton MN, Cooper KH, editors. Studies on the Ecology of Maputaland: Rhodes University and Natal Branch of the Wildlife Society of Southern Africa, Durban. pp. 1–7.

[23] Balinsky BI (1967) On some intrinsic and environmental factors controlling the distribution of dragonflies (Odonata) with redescription and a new name for a little-known species. Journal of the Entomological Society of Southern Africa 29: 3–22.

[24] Balinsky BI (1961) Observations of the dragonfly fauna of the coastal region of Zululand, with the description of three new species. Journal of the Entomological Society of Southern Africa 24: 72–91.

[25] Smith J, Samways MJ, Taylor S (2007) Assessing riparian quality using two complementary sets as bioindicators. Biodiversity Conservation 16: 2695–2713.

[26] Simaika JP, Samways MJ (2008) Valuing dragonflies as service providers. In: Córdoba-Aguilar A, editor. Dragonflies and Damselflies: Model Organisms for Ecological and Evolutionary Research. Oxfor University Pres, Oxford, UK. pp. 109–123.

[27] Samways MJ (2008) Dragonflies and damselflies of South Africa. Bulgaria: Pensoft.

[28] Simaika JP, Samways MJ (2008) Valuing dragonflies as service providers. In A. Cordoba-Aguilar (ed.). Dragonflies: Model Organisms for Ecological and Evolutionary Research, pp. 109–123, Oxford University Press, Oxford.

[29] Simaika JP (2008) Conservation Biogeography of Southern African Dragonflies (Odonata), MSc Thesis, Stellendosch University.

[30] Simaika JP, Samways MJ (2012) Using dragonflies to monitor and prioritize lotic systems: a South African perspective. Organisms, Diveristy & Evolution 12: 251–259.

[31] Pinhey E (1985) A survey of the dragonflies (Odonata) of South Africa, 2. Journal of the Entomological Society of Southern Africa 48: 1–48.

[32] Pinhey E (1984) A survey of the dragonflies (Odonata) of South Africa, 1. Journal of the Entomological Society of Southern Africa 47: 147–199.

[33] Samways MJ (2006) National Red List of South African Odonata. Odonatologica 35: 341–368.

[34] Balinsky BI (1965) A preliminary list of dragonflies (Odonata) of the Kruger National Park. Koedoe 8: 95–96.

[35] Clark TE, Samways MJ (1994) An inventory of the damselflies and dragonflies (Odonata) of the Kruger National Park, with three new South African records. African Entomology 2: 61–64.

[36] Simaika JP, Samways MJ, Kipping J, Suhling F, Dijkstra K-DB, et al (2013) Continental-scale conservation prioritization of African dragonflies. Biological Conservation 157: 245–254.

[37] Simaika JP, Samways MJ (2009) An easy-to-use index of ecological integrity for prioritizing freshwater sites and for assessing habitat quality. Biodiversity and Conservation 18: 1171–1185.

[38] Samways MJ, Grant PBC (2007) Honing red list assessments of lesser-known taxa in biodiversity hotspots. Biodiversity and Conservation 16: 2575–2586.

[39] Magoba RN, Samways MJ (2010) Recovery of benthic macroinvertebrate and adult dragonfly assemblages in response to large scale removal of riparian invasive alien trees. Journal of Insect Conservation 14: 627–636.

[40] Harabiš F, Dolný A (2012) Human altered ecosystems: suitable habitats as well as ecological traps for dragonflies (Odonata): the matter of scale. Journal of Insect Conservation 16: 121–130.

[41] Simaika JP, Samways MJ (2011) Comparative assessment of indices of freshwater habitat conditions using different invertebrate taxon sets. Ecological Indicators 11: 370–378.

[42] Samways MJ (2010) Impacts of extreme weather and climate change on South African dragonflies. BioRisk 5: 73–84.

[43] Oertli B, Joye DA, Castella E, Juge R, Cambin D, et al (2002) Does size matter? The relationship between pond area and biodiversity. Biological Conservation 104: 59–70.

[44] Kati V, Devillers P, Dufrene M, Legakis A, Vokou D, et al (2004) Testing the value of six taxonomic groups as biodiversity indicators at a local scale. Conservation Biology 18: 667–675.

[45] Bruton MN (1980b) Conservation and development in Maputaland. In: Bruton MN, Cooper KH, editors. Studies on the Ecology of Maputaland: Rhodes University and Natal Branch of the Wildlife Society of Southern Africa, Durban. pp. xvii-xix.

