# Supplementary material for: Species Composition, Distribution and Habitat Types of Odonata in the iSimangaliso Wetland Park, KwaZulu-Natal, South Africa and the Associated Conservation Implications
Source: PLoS One. 2014 Mar 24;9(3):e92588. doi: 10.1371/journal.pone.0092588 (PMC3963920; doi:10.1371/journal.pone.0092588)
Supplement: Appendix S1 — Odonata species account for iSimangaliso Wetland Park. Nine families are arranged in taxonomic order, with species accounts appearing alphabetically. (DOC) [file pone.0092588.s001.doc]

**Supporting Information**

**Appendix S1** Odonata species account for iSimangaliso Wetland Park. Nine families are arranged in taxonomic order, with species accounts appearing alphabetically.

**Family CALOPTERYGIDAE Demoiselles**

*Phaon iridipennis* GLISTENING DEMOISELLE (caught, photographed). Locally common in forest understorey near water. EKZNW database lists eight specimen records, from Mkhuze, Nyalazi, Sibaya, St Lucia and Kosi. We recorded it commonly at Kosi in Apr 2004 and it was present on the Mkuze River in numbers in Dec 2009 (>50 males at Site 18) but scarce at the same site in Feb 2011 (one counted).

**Family CHLOROCYPHIDAE Jewels**

*Platycypha caligata* DANCING JEWEL. Locally common on forested streams and rivers. EKZNW database has two records from Nyalazi River and Msunduzi River in Mkhuze. We recorded its presence at Kosi in Apr 2004 and counted 21 males at three other sites (10 at Samango Crossing, Dec 2009; 11 on two sites along the Mkuze River, Feb 2011).

**Family COENAGRIONIDAE Sprites**

*Aciagrion dondoense* OPAL SLIM (we did not record this species). No records in EKZNW database. The only recorded occurrence of this species from South African is from Mfabeni Swamp where it was abundant in Feb 2001 but has not since been present (Samways 2006). Red-listed as being of “least concern” by Samways (2006), where is referred to as *Aciagrion cf zambeziense*.

*Africallagma glaucum* SWAMP BLUET (we did not record this species). Abundant on any swampy ground in the interior of South Africa but seemingly rare in coastal Zululand. EKZNW database lists three records from E and W Shores.

*Agriocnemis exilis* LITTLE WHISP (caught, photographed). Locally abundant on grassy pans. EKZNW database lists four records, one from Nyalazi River, St Lucia 1957 (Pinhey 1984), others from E Shores, Sibaya and Kosi. We found it to be common on a grassy pan at Kosi Bay (Site 1) and present at two sites on E & W Shores.

*Agriocnemis falcifera* WHITE-MASKED WHISP (we did not record this species). Localised on grassy pans. One 1997 record from Lake Ngobezeleni in EKZNW database. Collected by Balinsky at Lake Sibaya in Dec 1964 (in collection at National Museum, Pretoria). The distribution map in Samways (2008) plots its presence at Lake Sibaya.

*Agriocnemis gratiosa* GRACIOUS WHISP (we did not record this species). Localised on grassy pans. EKZNW database has one record from Vidal and the distribution map in Samways (2008) plots its presence on E Shores. Red-listed as being of “least concern” by Samways (2006).

*Agriocnemis ruberrima* subspecies *ruberrima* ORANGE WHISP (caught, photographed). Scarce, localised on grassy pans. EKZNW database six records, all pre-1995, from E Shores between St Lucia and Lake Bhangazi. First described in 1961 (Balinsky 1961), from the type collected at Richard’s Bay in Dec 1957. We recorded it twice – one female at Site 37 (E Shores) and >20 at Kosi (Site 1) in a grassy pan. Red-listed as being “endangered” by Samways (2006).

*Azuragrion nigridorsum* BLACK-TAILED BLUET (caught, released). Locally abundant on grass- and sedge-fringed still-water ponds. EKZNW database uses the genus name *Enallagma* and lists 18 records from St Lucia to Kosi. We found it in abundance at one site (50 males counted at Site 27, Mkhuze) in Dec 2009, but none there in Feb 2011. We counted another 14 males at three other sites; none recorded on E Shores.

*Ceriagrion glabrum* COMMON CITRIL (caught, released). Abundant and widespread on grassy pans. EKZNW database lists 25 records from St Lucia to Kosi Bay. We caught and examined numerous individuals across the park to confirm identity (confirming identity as *glabrum,* not *suave*) and counted 190 individuals at 31 sites; most abundant at Site 49 (31 counted, Hippo Pan, W Shores).

[*Ceriagrion suave* SUAVE CITRIL (we did not record this species). EKZNW database lists one record for Kosi: this is of a female collected in July 1948 where Pinhey (1984) states “identification needs confirmation”. The record is not plotted on the distribution map in Samways (2008) and it is recommended that this record be rejected until confirmation is obtained.]

*Ischnura senegalensis* AFRICAN BLUETAIL (caught, released). Locally abundant on margins of still-water ponds, pans, and dams; tolerant of saline water and high algal levels. EKZNW database has 16 records from Maphelana to Kosi. We counted 107 individuals at 16 sites; most common on W Shores, with 46 counted on one pan with a large population of hippo (Site 49).

*Pseudagrion acaciae* ACACIA SPRITE. Locally common on wide, open rivers at low altitudes. EKZNW database has one record from Mkhuze. We only recorded it on the Mkuze River (Site 13) and counted two males here in Feb 2011.

*Pseudagrion coeleste* (subspecies *umsingaziense*) UMSINGAZI SPRITE (caught, photographed). Locally common on open still-water pans and ponds covered with *Nymphaea*. EKZNW database (where it is listed by its earlier name *Pseudagrion umsingaziense*) has no records for iWP. Red-listed as “vulnerable” by Samways (2006). We counted 57 males at 7 sites from Mkhuze to Kosi but did not record it on E or W Shores; most abundant at Neshe Pan (33 counted here in Feb 2011).

*Pseudagrion commoniae* BLACK SPRITE (caught, released). Locally common on ponds and sluggish river edges at low altitudes. EKZNW database has three records from Mkhuze, Nyalazi River and Sibaya. We found it present at only two sites, both in Mkhuze and counted nine males (Sites 14 & 19).

*Pseudagrion hageni* HAGEN’S SPRITE (caught, released). Locally common on forested streams under a closed canopy. EKZNW database has eight records from St Lucia to Kosi. We counted a total of 11 males at two sites at Kosi and one site on W Shores (Mpathi stream, Site 47). At Samango Crossing in Dec 2009 we counted 20.

*Pseudagrion hamoni* HAMON’S SPRITE (caught, released). Locally common on open still-water pans and ponds. EKZNW database has one record from Mkhuze. We counted 20 males at six sites in Mkhuze in Dec 2009 and Feb 2011 and recorded its presence at Kosi in Apr 2004.

*Pseudagrion kersteni* KERSTEN’S SPRITE (caught, released). Locally abundant on grass-fringed streams in the interior, largely absent from coastal plain. No records in EKZNW database. We caught one female at Mkhuze on outlet stream from dam (Site 27) in Dec 2009 and recorded its presence at Kosi in Apr 2004.

*Pseudagrion massaicum* MASAI SPRITE (caught, released). Common and widespread on open still-water pans and ponds, especially those covered with *Nymphaea*. EKZNW database has three records from St Lucia to Sibaya. We counted 62 at 12 sites; it was most abundant on Neshe Pan where 31 males were counted; curiously none were found on E or W Shores.

*Pseudagrion sublacteum* CHERRY-EYE SPRITE (caught, released). Locally common on flowing rivers. EKZNW database has one record from Mkhuze. We recorded it at two sites in Mkhuze, and counted five males here in Dec 2009, seven in Feb 2011.

**Family PLATYCNEMIDIDAE Featherlegs**

*Elattoneura glauca* COMMON THREADTAIL (caught, released). Localised along forested streams. EKZNW database has one record from Kosi. We counted six males at two sites, both on forested streams (Sites 2, 7) in Feb 2011 and Dec 2009 respectively.

**Family LESTIDAE Spreadwings**

*Lestes pallidus* PALLID SPREADWING (caught, released). Localised, on grassy pans. No records in EKZNW database. We counted six males at small rain-filled pans and a dam at Mkhuze (Sites 10, 11, 25, 27) in Dec 2009; none present there in Feb 2011.

*Lestes tridens* SPOTTED SPREADWING (caught, released). Localised, on grassy pans. EKZNW database has 10 records from St Lucia to Kosi. We recorded it commonly at Kosi in Apr 2004 and counted seven males at Mkhuze (Site 27) in Dec 2009.

*Lestes uncifer* SICKLE SPREADWING. (we did not record this species). Localised, on grassy pans. EKZNW database has one record from Mfabeni Swamp near Cape Vidal.

**Family GOMPHIDAE Clubtails**

*Ictinogomphus ferox* COMMON TIGERTAIL (caught, released). Common along margins of open waterbodies, especially large pans. EKZNW database has 12 records from Maphelana to Kosi, 1974 to 2001. We recorded it widely and counted 58 individuals at 12 sites. At Nsumo Pan in Mkhuze it was absent in Dec 2009 but plentiful in Feb 2011.

*Paragomphus cognatus* ROCK HOOKTAIL (caught, released). An common gomphid in the interior of South Africa but seemingly rare in coastal Zululand. No records in the EKZNW database. We recorded one female at Samango Crossing (Site 7) in Dec 2009 and one female at Kosi in Apr 2004.

*Paragomphus genei* GREEN HOOKTAIL (caught, photographed). Locally common at wooded margins of rivers and ponds. EKZNW database has four records from Cape Vidal to Sibaya. We counted nine at two sites (six at Site 38, E Shores, three at Lake Sibaya, Site 6) in Feb 2011.

[*Phyllogomphus selysi* (previously *brunneus*) BOLD LEAFTAIL (we did not record this species). EKZNW database (where it is listed by former name) has one record from Mkhuze in 1995. This record is not included in Samways’ (2008) and on this basis we recommend that the record be rejected until confirmation obtained].

**Family AESHNIDAE Hawkers, Emperors**

*Zosteraeschna minuscula* FRIENDLY HAWKER (we did not record this species). Common on streams and ponds in interior, largely absent from the coastal plain. One undated record from E Shores (Samways 2008). No records in EKZNW database.

*Anaciaeschna triangulifera* EVENING HAWKER (caught, released). A crepuscular species found hawking in flocks over woodland near water. EKZNW database has four records from Maphelana to Sodwana, 1984 to 2000; one collected at Dukuduku forest at a night light by N Duke in Jan 1984 (Pinhey 1984). We recorded this species at dusk at Kosi in Apr 2004.

*Anax ephippiger* VAGRANT EMPEROR (caught, photographed). Widespread and abundant at times in northern South Africa. EKZNW database records this species as *Hemianax ephippiger*, and has four records from Maphelana to Kosi, 1992 to 2000. We counted 24 at nine sites, with nine the largest number counted together at a grassy pan (Site 28) on E Shores.

*Anax imperator* BLUE EMPEROR (caught, released). Common and widespread across South Africa, breeding on rivers, pans, dams, and vleis. EKZNW database has 13 records from Maphelana to Kosi, 1980 to 2001. We counted 16 at nine sites.

*Anax speratus* ORANGE EMPEROR. Locally common on interior rivers, rare on coastal plain. No records in the EKZNW database. We had sight records of two males on river inlet to 4th Lake on Kosi (Site 5) in Feb 2011.

*Anax tristis* BLACK EMPEROR. Scarce on seasonal vleis in savanna; has a high incidence of vagrancy. No records in the EKZNW database. We recorded two flying individuals at St Lucia village in Jan 2006 (one at Crocodile Farm, one at Sewage Works).

*Gynacantha manderica* LITTLE DUSK-HAWKER (caught, photographed). Scarce in lowland and riparian forest. No records in the EKZNW database. We recorded relatively large numbers (30 counted) at two sites in Mkhuze (Sites 17, 18) in Dec 2009 but none present here in Feb 2011.

*Gynacantha usambarica* (previously *zuluensis*) USAMBARA DUSK-HAWKER (caught, photographed). Restricted to coastal forest; easily overlooked and possibly common. EKZNW database (where it is listed by former name) has three records from St Lucia to Kosi, 1984 to 2000. We recorded four individuals at Kosi in Apr 2004 and one in Feb 2011.

*Gynacantha villosa* HAIRY DUSK-HAWKER (we did not record this species). Rare (or overlooked) in coastal forest. EKZNW database has a single record from Charter’s Creek where one was caught in a malaise trap by J Londt in Oct 1977 (Pinhey 1984). There is only one other South African record, from Richards Bay where it was collected by Balinsky in Dec 1957 (Pinhey 1984). Red-listed as being of “least concern” by Samways (2006).

**Family CORDULIIDAE Presbas, Emeralds**

*Hemicordulia africana* AFRICAN EMERALD (caught, photographed). Scarce, restricted to shaded pools in swamp

forest. EKZNW database records this species as *Hemicordulia asiatica*, and has three records from Dukuduku to Kosi, 1984 to 2000. We recorded two individuals in swamp forest at Kosi in Apr 2004.

*Phyllomacromia contumax* TWO-BANDED CRUISER. A solitary species spending much of day on the wing in savanna/wetland mosaics. EKZNW database records this species as *Macromia bifasciata*, and has 13 records from St Lucia to Kosi, 1926 to 2004. We had sight records of three individuals, one at Kosi (Apr 2004), one at Mkhuze (Site 27, Dec 2009), one at Sibaya (Site 6, Feb 2011).

*Phyllomacromia picta* DARTING CRUISER. A solitary species spending much of the day on the wing, especially along wooded rivers. No records in the EKZNW database. We had sight records of two individuals, one in Mkhuze at Site 26 (Dec 2009) and one at Site 13 (Feb 2011).

**Family LIBELLULIDAE Skimmers, etc**

*Acisoma panorpoides* PINTAIL (caught, released). Locally abundant in seasonally flooded vleis in savanna. EKZNW database has seven records from St Lucia to Kosi. We counted 88 individuals at nine sites; it was especially plentiful (65 counted) at a freshwater seep bordering Lake St Lucia, on E Shores (Site 37).

*Aethriamantra rezia* PYGMY BASKER (caught, photographed). A localised, tropical species frequenting reed- and sedge-lined ponds and pans. EKZNW database has five records from St Lucia to Sodwana, 1981 to 2001. We recorded it at two sites: 1female in flooded grass on E Shores (Site 39) and three males at the river inlet on Kosi Bay’s 4th Lake (Site 5).

*Brachythemis leucosticta* BANDED GROUNDLING (caught, released). Abundant throughout savanna areas, frequenting open water-bodies that have bare muddy margins. EKZNW database has 24 records from Maphelana to Kosi, 1974 to 2002. It was the single most common odonate that we encountered with literally thousands present at some localities. We counted 920 individuals at 25 sites; in Mkhuze numbers varied greatly from 189 in Feb to 696 in Dec.

*Bradinopyga cornuta* DON-DWALA (caught, photographed) Localised, frequenting streams and rivers that are flanked by rock faces on which it clings. No records in the EKZNW database. We caught and photographed one near Mantuma Camp in Mkhuze in Feb 2010.

*Chalcostephia flavifrons* INSPECTOR (caught, photographed). Localised, restricted to shaded ponds in forest. EKZNW database has 10 records from St Lucia to Kosi, 1974 to 2001. We counted 22 individuals at eight sites.

*Crocothemis erythraea* BROAD SCARLET (caught, released). Abundant and widespread on grass and sedge fringes of rivers, pans, dams and floodplains. EKZNW database has 12 records from Maphelana to Kosi, 1991 to 2004. The most widely occurring species in the park; we counted 204 individuals at 28 sites.

*Crocothemis sanguinolenta* LITTLE SCARLET (we did not record this species). Common and widespread on still water-bodies in the interior, but seemingly absent from the EKZNW coastal plain. EKZNW database has one record from Mkhuze, 1991.

*Diplacodes lefebvrii* BLACK PERCHER (caught, photographed). Common and widespread in grass- and sedge-margins of shallow bodies of open water. EKZNW database has 22 records from Maphelana to Kosi, 1979 to 2005. We counted 114 individuals at 18 sites, with concentrations on several grassy pans on E Shores (Site 33, 41, 43).

*Diplacodes* (previously *Philonomon*) *luminans* BARBET (caught, released). Common on seasonally flooded vleis in savanna. EKZNW database (where it is listed by former name) has four records from E Shore to Kosi from 1974 to 1999. We counted 147 males at 12 sites; particularly common (48 counted) in recently-flooded grass (Site 33) on E Shores in Feb 2011; 40 counted at Umkhumbi Dam, Mkhuze (Site 27), in Dec 2009, none here in Feb 2011.

*Diplacodes pumila* (previously *deminuta*) DWARF PERCHER (caught, photographed). Rare, localised, on grassy pans. EKZNW database (where it is listed by former name) has two records, one from Mfabeni Swamp, E Shores (1990) and one collected at Lake St Lucia in Mar 1974 by F de Moor (Pinhey 1984). Red-listed as being of “least concern” by Samways (2006) and stated here that there is currently no known population in South Africa. We recorded two males alongside *lefebvrii* on E Shores at Site 33.

*Hemistigma albipunctum* PIED-SPOT (caught, photographed). Very common in seasonally flooded vleis in savanna. EKZNW database has 19 records from Maphelana to Kosi. We counted 280 individuals at 21 sites, particularly common (93 counted) in flooded grass and sedge (Site 39) on E Shores.

*Macrodiplax cora* CORA’S PENNANT (we did not record this species). Offshore vagrant with a high tolerance for saline water. EKZNW database has one undated record; it refers to two specimens collected (and many more seen) on the shores of Kosi Bay lagoon in Dec 1964 (Balinsky’s 1967). This is the first-recorded occurrence of this Asian species on the African continent; in Feb 2007 another female was photographed at the St Lucia estuary by Paul Schrijvershof (www.africa-dragonfly.net); these are the only currently recorded South African records.

*Nesciothemis farinosa* BLACK-TAILED SKIMMER (caught, photographed). Common and widespread along grass- or sedge-lined margins of pans and rivers. EKZNW database has 12 records from St Lucia to Kosi, 1974 to 2004. We counted 58 individuals at 16 sites.

*Notiothemis jonesi* FOREST-WATCHER. Localised and easily overlooked, frequenting small, still-water ponds under forest canopy. No records in the EKZNW database. We found one in the Mkhuze Fig Forest (Site 18) in Feb 2011.

*Orthetrum abbotti* ABBOTT’S SKIMMER (caught, photographed). Localised on grassy fresh-water seeps. EKZNW database has one record from St Lucia in 1990. We counted 18 at two sites on E Shores.

*Orthetrum chrysostigma* EPAULET SKIMMER (we did not record this species). Common and widespread in the interior on rivers, dams and pans. EKZNW database has five records from St Lucia, Charter’s Creek, Mkhuze, 1990 to 2002.

*Orthetrum hintzi* HINTZ’S SKIMMER (caught, photographed). Locally common on grassy pans in savanna. EKZNW database has two records, one from St Lucia estuary in Jan 1984 (Pinhey 1984), one from 1990. We counted four individuals in Feb 2011 on E Shores, and one at Samango Crossing (Site 7) in Dec 2009.

*Orthetrum icteromelas* SPECTACLED SKIMMER (caught, photographed). Locally common on grassy pans in savanna. EKZNW database has eight records from St Lucia to Kosi, 1984 to 2001. We counted six individuals at four sites, most on E Shores.

*Orthetrum julia* JULIA SKIMMER (caught, photographed). Common along wooded and forested rivers and streams. EKZNW database has 14 records from Maphelana to Kosi, 1994 to 2001. We counted 57 at 14 sites; highest numbers (20) were counted at Samango Crossing (Site 7) in Dec 2009.

*Orthetrum machadoi* MACHADO’S SKIMMER (we did not record this species). Locally abundant in savanna floodplains in the interior, apparently rare on coastal plain. EKZNW database has one record from St Lucia, collected Dec 1957 (Pinhey 1984).

*Orthetrum robustum* ROBUST SKIMMER (caught, photographed). Restricted, in South Africa, to coastal plain on open water-bodies with short grassy verges. EKZNW database has 10 records from St Lucia to Kosi, 1984 to 2001. Type was described by Balinsky (1965) from specimens collected at Richards Bay in Dec 1957; We recorded in present at Kosi in Apr 2004 and counted 27 at three sites on E Shores, most (20) on Mfabeni Swamp; not recorded at Mkhuze or Kosi.

*Orthetrum stemmale* STRONG SKIMMER (caught, photographed). Localised on open water-bodies. EKZNW database has two records from St Lucia from 1975 and 1979 (incorrectly recorded as *Orthetrum brachiale*). We recorded 14 of this species at six sites in Mkhuze in Dec 2009, but none there in Feb 2011.

*Orthetrum trinacria* LONG SKIMMER (caught, photographed). Common, widespread on large open water-bodies, especially those with bare, muddy edges; more tolerant of saline water than most odonata. EKZNW database has 14 records from Maphelana to Kosi, 1948 to 2001. We counted 28 at 15 sites.

*Palpopleura jucunda* YELLOW-VEINED WIDOW (caught, released). Locally common in damp freshwater grass and sedge seeps. No records in the EKZNW database. We found it to be numerous (16 counted) at a just one locality, a freshwater seep bordering Lake St Lucia, on E Shores (Site 37)

*Palpopleura lucia* LUCIA WIDOW (caught, released). Common throughout savanna areas on open sedge- and grass-filled pans. EKZNW database does not distinguish this species from *Palpopleura portia* and lists 20 records of *lucia/portia* from Maphelana to Kosi, 1936 to 2000. We found it outnumbered *portia* 15:1, counting 126 males at 12 sites; it was most numerous on the dams at Sites 26 and 27 in Mkhuze.

*Palpopleura portia* PORTIA WIDOW (caught, released). EKZNW database does not distinguish this species from *Palpopleura lucia* (see previous species). We counted seven males at two sites on E Shores (Sites 39, 40); it was not recorded elsewhere; *lucia* was absent from both these sites.

*Pantala flavescens* PANTALA (caught, photographed). An abundant, wide-ranging species across South Africa. EKZNW database has eight records from Maphelana to Kosi, 1974 to 2001. We counted 276 individuals at 26 sites; it was very numerous on E Shores but only encountered in small numbers elsewhere. We estimated 130 individuals in a group at Site 36 on E shores.

*Parazyxomma flavicans* BANDED DUSK-DARTER (photographed). Scarce, or perhaps overlooked; crepuscular, hiding in riparian forest during the day. No records in the EKZNW database. We recorded two at Mkhuze (Sites 16, 22) in Dec 2009.

*Rhyothemis semihyalina* PHANTOM FLUTTERER (caught, released). A common savanna species with widespread vagrancy. EKZNW database has 16 records from Maphelana to Kosi, 1974 to 2000. We counted 88 at 15 sites in 2011 and 10 at five sites in Mkhuze in 2009; it was especially numerous at the 4th Lake inlet at Kosi (Site 5, 32 counted).

*Sympetrum fonscolombii* NOMAD (we did not record this species). Common in the interior, apparently scarce on the coastal plain. EKZNW database has four records, all from Mkhuze, 1991 to 2005.

*Tetrathemis polleni* BLACK-SPLASH (photographed). Locally common on shaded streams and pools in forest. EKZNW database has six records from Maphelana to Kosi, 1959 to 2001. We recorded 22 males at five sites in 2011 and seven at two sites in Mkhuze in 2009.

*Tholymis tillarga* TWISTER (photographed). A localised tropical savanna species, crepuscular, hiding in shrubbery during the day, breeding on open water. EKZNW database has one record from Feb 1984 at St Lucia, seen there by Duke & Pinhey (Pinhey 1984). We recorded a total of four males at Sites 31 and 38 on E Shores, hiding in low foliage during the day.

*Tramea basilaris* KEYHOLE GLIDER (caught, released). Very common and wide-ranging in open savanna, breeding on any open water. EKZNW database records this species as *Trapezostigma basilare*, and has 12 records from St Lucia to Kosi, 1926 to 2000. We counted 143 individuals at 27 sites; it was especially numerous on E Shores (127 of the 143 records from here).

*Tramea limbata* FERRUGINOUS GLIDER (caught, released). Wide-ranging in open savanna, breeding on any open water. EKZNW Wildlife database records this species as *Trapezostigma continentale*, and has nine records from Maphelana to Kosi, 1926 to 2001. We counted four individuals at two sites on E Shores.

*Trithemis aconita* MONKSHOOD DROPWING. Scarce, localised along forested streams. No records in the EKZNW database. We recorded several males along a forested stream (Site 4) at Kosi Bay in Apr 2004; not recorded in 2009 or 2011.

*Trithemis annulata* VIOLET DROPWING (caught, released). A savanna species, common along reed- and sedge-lined margins of open still water. EKZNW database lists 11 records from E Shores to Kosi, 1974 to 2001. We counted 46 males at 11 sites; it was most numerous at Mkhuze (25 at five sites) and least numerous on E Shores (seven at two sites).

*Trithemis arteriosa* RED-VEINED DROPWING (caught, released). Abundant on ponds and streams in the interior, less common on the coastal plain. EKZNW database lists 11 records from St Lucia to Kosi, 1974 to 2001. We counted 64 males at 13 sites, most of these (49) in Mkhuze.

*Trithemis dorsalis* DORSAL DROPWING (we did not record this species). Common along rivers in the interior but largely absent from the coastal plain. EKZNW database lists one record for Mkhuze from 1994.

*Trithemis furva* NAVY DROPWING (caught). Common in the interior, scarce on the coastal plain; along vegetated margins of flowing rivers and streams. EKZNW database lists one record for Sodwana Bay from 1973. We recorded three at Samango Crossing (Site 7) in Dec 2009.

*Trithemis hecate* HECATE DROPWING (caught, photographed). A savanna species, locally common on open grassy pans. EKZNW database lists three records from E Shores, in 1990 and 2001, and recorded there by Duke & Pinhey in Jan and Feb 1984 (Pinhey 1984). We recorded many individuals on a grassy pan at Kosi (Site 1) in Apr 2004 but did not find it there or elsewhere in 2009 or 2011.

*Trithemis kirbyi* KIRBY’S DROPWING (caught, released). Common and widespread on still and flowing waters in the interior but largely absent from the EKZNW coastal plain. EKZNW database lists three records, one from 1974 (collected at Sodwana in 1973 according to Pinhey 1984), two from Mkhuze in 1994 and 2001. We recorded a total of four males in Mkhuze at sites 15, 17, 18.

*Trithemis pluvialis* RIVER DROPWING (we did not record this species). Locally common along vegetated margins of fast-flowing streams. EKZNW database has one record from Nyalazi River, Dec 1957 (Pinhey 1984).

*Trithemis stictica* JAUNTY DROPWING (caught, released). Common in the interior along vegetated fringes of slow-flowing streams and rivers; apparently scarce on the EKZNW coastal plain. EKZNW database has seven records from St Lucia to Kosi, 1989 to 2000. We recorded 3males on a forested stream (Site 2) at Kosi.

*Urothemis assignata* RED BASKER (caught, released). A savanna species, common on margins of open pans. EKZNW database has four records from St Lucia area, 1936 to 1997. We counted 47 males at 13 sites; was especially numerous at Mfabeni Swamp (Site 31, 13 counted).

*Urothemis edwardsii* BLUE BASKER (caught, photographed). A savanna species, common on reed- or sedge-lined margins of large, open water-bodies. EKZNW database has eight records from Kosi, Sibaya, Mkhuze and E Shores, 1959 to 2001. We counted 36 males at seven sites; it was especially numerous at Site 5 (25 counted) at Kosi in Feb 2011.

*Urothemis luciana* ST LUCIA BASKER (we did not record this species). Rare, erratic, on open grassy pans. EKZNW database has six records, four from Balinsky from W Shores (Dec 1957, Dec 1959), one from Samways (2006, Kosi Bay Dec 2000) and one apparently in error from Maputa dated Nov 1945. Red-listed as being of “least concern” by Samways (2006); Type described by Balinsky (1961) from specimens collected on western shore of Lake St Lucia in Dec 1957 and Dec 1959; not recorded again until Dec 2000 at Kosi Bay (Samways 2006).

*Zygonyx torridus* RINGED CASCADER (we did not record this species). Restricted to fast-flowing rivers at cascades. EKZNW database has two records from Mkhuze from 1991 and 8 Feb 2000.

*Zyxomma atlanticum* LITTLE DUSK-DARTER (caught, photographed). Localised in swamp forest around deeply shaded ponds. EKZNW database has two records from Kosi Bay collected on 19 Feb 1999 and 27 Feb 2001; Balinsky collected it at Kosi Bay in Dec 1964. We recorded several at Charters Creek in Mar 2002 and at Kosi Bay in Apr 2004 and Feb 2011.
